# Cardiovascular screening in rheumatoid arthritis: a cross-sectional primary care database study

**DOI:** 10.1186/1471-2296-14-150

**Published:** 2013-10-10

**Authors:** Helen L Monk, Sara Muller, Christian D Mallen, Samantha L Hider

**Affiliations:** 1 Arthritis Research UK Primary Care Centre, Primary Care Sciences, Keele University, Keele, UK

## Abstract

**Background:**

Patients with rheumatoid arthritis (RA) are known to be at increased risk of vascular disease. It is not known whether screening for vascular risk factors occurs in primary care. The aim of this study was to determine whether guidance advocating cardiovascular screening in RA patients is being implemented in primary care.

**Methods:**

This study was undertaken in a UK primary care consultation database. All patients with a diagnosis of RA between 2000 and 2008, and still registered with the GP practice in 2009 were matched by age, gender and GP practice to three non-RA patients. Evidence of screening for five traditional vascular risk factors (blood pressure, lipids, glucose, weight, smoking) was compared in those with and without RA using logistic regression models. A comparison was also made with diabetes.

**Results:**

401 RA patients were identified and matched to 1198 non-RA patients. No differences in the overall rates of screening were found (all five risk factors: RA 24.9% vs no RA 25.6%), but RA patients were more likely to have a smoking status recorded (67% versus 62%). In contrast, those with diabetes were up to 12 times as likely to receive vascular screening.

**Conclusions:**

Despite the excess risk of vascular disease in patients with RA being of a similar magnitude to that seen in diabetes, patients with RA did not receive additional CVD screening in primary care, although this was achieved in patients with diabetes. More emphasis needs to be placed on ensuring those with RA are actively screened for cardiovascular disease in primary care.

## Background

It is well recognised that patients with rheumatoid arthritis (RA) have an increased mortality rate compared to the general population, due mainly to an excess risk of cardiovascular disease (CVD) [1-3]. This is reflected in both the European League Against Rheumatism (EULAR) and British Society for Rheumatology guidelines, which recommend regular CVD screening and aggressive risk factor management in patients with RA [4,5]. However, it is recognised that traditional CVD risk assessment tools, such as the Framingham score, substantially underestimate the risk of cardiovascular disease in patients with RA [6]. To mitigate this EULAR guidelines [5] recommend an adjustment by a factor of 1.5 in the presence of specific criteria (disease duration greater than 10 years, rheumatoid factor or anti-cyclic citrullinated peptide positivity and the presence of extra-articular manifestations of RA) when traditional CVD screening tools are used. However, the work of Crowson et al. [6] suggests that at older ages, even an adjustment factor of 1.5 may be insufficient. QRISK2 is the only validated CVD risk assessment tool to include RA as an independent risk factor for CVD [7], and indeed other UK guidance from the Joint British Societies Guidance 2 [8] fails to mention rheumatoid arthritis, further contributing to the lack of awareness of the increased risk of CVD in patients with RA. Studies suggest that the excess CVD risk in RA is comparable to that seen in type 2 diabetes mellitus, with an approximate doubling of the risk of CVD over three years [9,10]. For patients with diabetes this has been translated into UK guidance advocating annual screening and management of CVD risk [11], further supported with vascular risk factor screening for patients with diabetes being incentivised as part of the UK General Practice Quality Outcomes Framework (QOF), ensuring high uptake of screening for these patients [12]. Although guidelines recommend CVD screening for patients with RA, few studies have addressed whether this is being implemented in routine clinical care or where this screening should occur. The primary aim of this study therefore was to determine CVD screening practice in RA and comparable non-RA patients in a UK-based primary care population prior to the introduction of an incentivised annual review, which includes an assessment of CVD risk. A secondary aim was to compare rates of screening in people with RA to such rates in people with diabetes mellitus, who have similarly increased levels of vascular risk and in whom such screening is incentivised in the UK.

## Methods

### Identification of RA and non-RA patients

Data were extracted from the Consultations in Primary Care Archive (CiPCA), an anonymised database of clinical information from nine general practices in Staffordshire, UK [13]. RA patients were identified using diagnostic Read codes. Read codes are a hierarchical coding structure that all family physicians in the UK use to record diagnoses, symptoms or procedures in electronic patient records. Rather than having an administrative purpose, these codes are used to record clinical care and improve its efficiency.

All patients with a diagnostic Read Code for RA in the CiPCA database between 2000 and 2008 and still registered with the practice in 2009 were identified. RA patients were matched by age, gender and general practice to three patients without an RA Read coded diagnosis. Non-RA patients had to have consulted in the year in which the matched RA patient was first recorded as having RA, and also still be registered with the practice in 2009. The practices that contribute to CiPCA are part of the Keele GP Research Partnership and are subject to regular cycles of training, assessment and feedback, which help to ensure that the quality of the data is high [14]. CiPCA has been shown to give similar prevalence estimates for musculoskeletal disease as other national databases [13].

### Ethical approval

Approval for download and research using these databases was gained from the North Staffordshire Research Ethics Committee (REC Reference: 03/04).

### Identification of screening and comorbidity

The CiPCA databases for 2009 were searched for Read codes indicating screening of five traditional CVD risk factors; blood pressure, lipid levels, smoking status, body weight or body mass index and blood glucose. These traditional CVD risk factors were selected as they are routinely identified and managed in primary care. Records for 2009 were also searched for morbidity codes and related prescriptions that indicated a diagnosis of diabetes mellitus, hypertension or hyperlipidaemia. We sought these diagnoses specifically, as they would increase future CVD screening. We took a cross-sectional approach to the use of data from 2009 only for analysis purposes. A list of Read codes used to identify RA patients, screening and comorbidities is available from the authors on request.

### Definition of screening outcomes

Although, in the UK, the National Institute for Health and Clinical Excellence recommends adults aged 40 to 74 years are screened for CVD, the priority for full risk estimation is those considered to be at high risk (10 year risk of CVD ≥20%). RA patients are also considered high risk [1-5]. Following the screening recommendations, CVD screening was defined in three ways: 1) “Any CVD screening” – screening for any one or more of the five factors; 2) “Standard CVD screening” – screening of blood pressure, smoking and lipids – these three factors were chosen since after accounting for age and gender, they are responsible for up to 80% of CVD risk in the general population [15]; and 3) “Comprehensive screening” – screening for all five factors.

### Statistical analyses

Basic demographic information was summarised using descriptive statistics, chi squared tests and t-tests to assess differences in screening between RA and non-RA patients as appropriate. The rates of primary care contacts (number of days with a contact with primary care) in 2009 were compared between those with and without RA. Logistic regression models, both unadjusted and adjusted for age and gender, were used to assess the association between each of the screening outcomes and RA status. Models were checked for the presence of correlation between patients within practices and formulated accordingly. The odds of screening in patients with RA and those with diabetes mellitus, hypertension and hyperlipidaemia were also compared using logistic regression. Results of all logistic regression analyses are presented as odds ratios with 95% confidence intervals. Statistical analyses were performed using Stata version 12.1 [16].

## Results

### Demographics of the study sample

401 RA patients were identified and successfully matched to 1198 controls (Table 1). As expected for an RA cohort 265 (66.1%) patients were female and the mean (standard deviation) age was 58.8 (12.7) years. The prevalence of diagnosed diabetes mellitus (12.0% vs. 10.6%, p=0.447) and hyperlipidaemia (33.4% vs. 33.2%, p=0.943) were very similar in RA and non-RA patients (Table 1). However, the prevalence of hypertension was significantly greater amongst RA patients (52.1% vs. 45.7%, p=0.025). In addition, RA patients had significantly more primary care contacts in 2009 than those without RA (18.9 vs. 11.4, p<0.001).

**Table 1:** Demographics of RA and Non-RA patients (n (%))

**Characteristic**	**RA (n=401)**	**Non-RA (n=1198)**	***P-value***
Female Gender	265 (66.1)	793 (66.2)	
Age groups (years)			
27-50	97 (24.2)	291 (24.3)	
51-65	181 (45.1)	543 (45.3)	
66-74	79 (19.7)	237 (19.8)	
75+	44 (11.0)	127 (10.6)	
Diabetes	48 (12.0)	127 (10.6)	0.447
Hypertension	209 (52.1)	547 (45.7)	0.025
Hyperlipidaemia	134 (33.4)	398 (33.2)	0.943
Mean number of contacts (SD)	18.9 (12.0)	11.4 (10.6)	<0.001

RA – Rheumatoid arthritis; SD – standard deviation.

### Rates of screening for cardiovascular risk factors

Blood pressure was the most commonly recorded risk factor amongst both RA and non-RA patients, with over 70% of patients having a blood pressure measurement (Table 2). Conversely, measurement of lipids was the risk factor least likely to be recorded amongst both groups (RA 45.9%; no RA 43.6%). The rate of screening was similar between RA and non-RA patients for all risk factors except smoking status, which was more likely to be recorded in patients with RA (67.1% vs. 61.6%, p=0.049) (Table 2).

**Table 2:** RA and non-RA patients screened for each cardiovascular risk factor (n (%))

**Risk factor**	**RA (n=401)**	**Non-RA (n=1198)**	***P-value***
Blood pressure	299 (74.6)	860 (71.8)	0.281
Lipids	184 (45.9)	522 (43.6)	0.420
Smoking status	269 (67.1)	738 (61.6)	0.049
Body weight	219 (54.6)	632 (52.8)	0.518
Glucose level	188 (46.9)	565 (47.2)	0.923

RA – Rheumatoid arthritis.

### Rates of CVD screening

There was no evidence of correlation of patients within GP practice and so single-level logistic regression models were employed. RA patients had greater odds of receiving 'any CVD screening’ than non-RA patients, even after adjustment for age, gender and comorbidity (OR 1.59 (1.13, 2.23)) (Table 3). This was due mainly to the increased recording of smoking status in those with RA. However the odds of receiving a standard (blood pressure, smoking and lipids) (1.01 (0.79, 1.29)) or comprehensive (all five factors) (0.96 (0.73, 1.25)) CVD screen were similar in RA and non-RA patients (Table 3).

**Table 3:** Association between RA status and receiving CVD screening

**Type of CVD screen**	**RA (n (%))**	**Non-RA (n (%))**	**Unadjusted (OR (95% CI))**	**Age and gender adjusted (OR (95% CI))**
Any	352 (87.8)	983 (82.1)	1.57 (1.13, 2.19)	1.59 (1.13, 2.23)
Standard	138 (34.4)	407 (34.0)	1.02 (0.80, 1.29)	1.01 (0.79, 1.29)
Comprehensive	100 (24.9)	307 (25.6)	0.96 (0.74, 1.25)	0.96 (0.73, 1.25)

CVD – cardiovascular disease; RA – Rheumatoid arthritis; OR – odds ratio; CI – confidence interval.

### The effect of comorbidity on standard CVD screening rates

The impact of other conditions and RA on rates of standard CVD screening was explored further by comparing the odds of screening in those with either one or both conditions compared to those with neither (Table 4). Individuals with diabetes without RA had over eleven times the odds of receiving a standard CVD screen (11.14 (6.86, 18.11)). However, the addition of RA in individuals with diabetes did little to alter their odds of screening (15.32 (6.70, 35.02)). A similar pattern was seen in relation to comprehensive screening (data not shown). Similar effects were seen with hyperlipidaemia although the effect was less marked for hypertension, in that the addition of RA failed to increase the odds of screening.

**Table 4:** The effect of comorbidity on standard CVD screening

	**Number of patients (n (%))**	**Unadjusted (OR (95% CI))**	**Age and gender adjusted (OR (95% CI))**
No RA or DM	1071 (67.0)	1.00	1.00
RA only	353 (22.1)	0.96 (0.74, 1.26)	0.96 (0.72, 1.24)
DM only	127 (7.9)	12.15 (7.53, 19.61)	11.14 (6.86, 18.11)
RA +DM	48 (3.0)	14.91 (6.62, 33.61)	15.32 (6.70, 35.02)
No RA or HTN	651 (40.7)	1.00	1.00
RA only	192 (12.0)	1.11 (0.74, 1.68)	1.16 (0.77, 1.76)
HTN only	547 (34.2)	5.34 (4.11, 6.94)	4.82 (3.67, 6.35)
RA +HTN	209 (13.1)	4.36 (3.11, 6.11)	3.82 (2.70, 5.42)
No RA or HLP	800 (50.0)	1.00	1.00
RA only	267 (16.7)	1.09 (0.76, 1.58)	1.07 (0.68, 1.66)
HLP only	398 (24.9)	12.31 (9.25, 16.38)	9.78 (6.98, 12.87)
RA +HLP	134 (8.4)	11.50 (7.62, 17.35)	8.15 (5.40, 12.31)

RA – Rheumatoid arthritis; HTN – hypertension; HLP – hyperlipidaemia; OR – odds ratio; CI – confidence interval.

## Discussion

This study found that patients with RA received similar rates of CVD screening as age, gender and practice matched non-RA patients, despite consulting more often in primary care. The exception to this was smoking status, which was more likely to be recorded in RA patients. In contrast, patients with a diagnosis of diabetes mellitus were significantly more likely to receive any, standard or comprehensive CVD screening. This suggests that although effective identification of high-risk patients with diabetes occurs in primary care and prompts screening, either RA patients are not recognised as high-risk or this increased risk is recognised but not translated into more CVD screening. Given that rates of primary care contact were greater amongst RA patients, our results cannot be explained by these patients receiving a large amount of contact with secondary care and a corresponding lack of contact with primary care.

There are a number of strengths and weaknesses that need to be considered when interpreting this study. Compared to existing studies of CVD screening in RA, with small secondary care populations (n<135) [17-19], ours is a large sample. The study also benefited from quality morbidity coding, ensuring that primary care screening and comorbidities were unlikely to be missed. Despite the relatively local nature of this database, previous work by Jordan et al. [13], suggests that local databases such as CiPCA are comparable to larger national databases. Also, although the CiPCA is known to provide high quality coding of clinical activity in primary care, it does not include a complete record of what happens to patients in secondary care. It is therefore possible that some RA patients are receiving CVD risk screening in secondary care, but this is not noted in their primary care record. However, if screening led to additional medications for diabetes, hyperlipidaemia or hypertension being introduced this would be included within primary care records. If significant secondary care screening is occurring but is not recorded, this is likely to improve with the addition of RA to the QOF component of the UK General Practice Contract, which took effect in April 2013.

In addition, although all patients had at least one diagnostic Read code for RA, we were unable to stratify patients by disease activity, severity or disease modifying anti-rheumatic drug (DMARD) treatment, as many of the hospital prescribed DMARDs such as parenteral methotrexate would not have been captured. Nevertheless the screening recommendations do not stratify RA in this way, and we do not expect that the recording of RA in primary care, or CVD screening as a result of an RA diagnosis, to be biased by the severity of disease. Hence, the lack of disease activity information is unlikely to account for the results. It is also possible that some patients with a single RA code did not actually have RA. This could have been avoided by the use of an algorithm for the diagnosis of RA (e.g. [20]). However, what is important in the primary care context is that patients that the GP considers to have RA are screened for CVD risk factors, and the use of a single RA code allows this to be tested.

This study is in keeping with existing work that has suggested that CVD screening is sub-optimal in RA patients [17-19,21-24] and perhaps also suggests that the omission of RA from CVD risk scores such as Framingham and the Joint British Societies may be masking the issue of CVD risk in patients with RA. Previous work also suggests that effective identification of, and incentives to screen, high risk groups can lead to changes in practice, e.g. [25].

There are several possible hypotheses as to why RA does not lead to additional CVD screening. First, it may be that the increased risk of CVD in RA is not recognised in primary care. In a study by Bell and Rowe [23], only 32% of family physicians identified RA as an independent risk factor for CVD, and only 15% assessed their patients for primary CVD prevention. Second, it may be that the excess CVD risk is recognised, but there is confusion over whose responsibility it is to screen for CVD, especially in complex RA patients, who may already be taking a number of medications or under regular secondary care follow up.

In common with the existing literature, the presence of comorbidity, especially diabetes was associated with screening [24,26], suggesting that despite the CVD risks being similar in RA and diabetes [10,11], screening practice is not: those with diabetes are significantly more likely to receive CVD screening. This may in part be explained by the higher population prevalence of diabetes (5.1% UK adults [27]) compared to RA (1% [28]), meaning the average family physician will manage significantly more patients with diabetes compared to those with RA. In addition, many of those with diabetes mellitus may be managed exclusively in primary care, and in the UK family physicians are incentivised to screen patients with diabetes for CVD under the Quality and Outcomes Framework (QOF) component of the General Practice Contract. This has been shown to lead to improved quality of diabetes care [12,13]. Similar quality indicators relating to CVD risk assessment have recently been introduced [29], in that RA patients aged between 30–85 will be expected to have had an annual CVD risk assessment, using a CVD risk assessment tool adjusted for RA. Given the effect of incentivising care on screening rates in diabetes it may be that these indicators will improve CVD screening rates for patients with RA, as might the recent introduction of NHS Health Checks for patients over the age of 40, especially if patients with RA are actively targeted.

## Conclusions

In summary, this study has shown that despite the wealth of literature and national guidelines, current practice for CVD screening of RA patients in primary care is suboptimal. This study suggests that more needs to be done to increase awareness in primary care physicians, nurses and patients regarding the excess CVD risk associated with RA and to agree how this excess risk should be managed. The inclusion of new incentives within the GP contract for cardiovascular screening in RA may lead to improved management for these patients.

## Competing interests

The authors declare no competing interests.

## Authors’ contributions

CDM and SH conceived the idea for and design of the study. HLM and SM carried out the analysis of the data. HLM drafted the manuscript. All authors were involved in the interpretation of data, critically revising the manuscript and approved the final version for submission.

## Pre-publication history

The pre-publication history for this paper can be accessed here:

http://www.biomedcentral.com/1471-2296/14/150/prepub
